# Proteomic analysis of plasma proteins during fentanyl withdrawal in ovariectomized female rats with and without estradiol

**DOI:** 10.1111/jne.70033

**Published:** 2025-04-20

**Authors:** Patricia Sinclair, Navdeep S. Dhanjal, E. Blair Towers, Wendy J. Lynch, Nadine Kabbani

**Affiliations:** ^1^ Interdisciplinary Program in Neuroscience George Mason University Fairfax Virginia USA; ^2^ Bioinformatics and Computational Biology Program George Mason University Manassas Virginia USA; ^3^ Department of Psychiatry and Neurobehavioral Sciences University of Virginia Charlottesville Virginia USA; ^4^ Medical Scientist Training Program University of Virginia Charlottesville Virginia USA; ^5^ School of Systems Biology George Mason University Manassas Virginia USA

**Keywords:** addiction, estrogen, opioid, withdrawal, women's health

## Abstract

Evidence from both clinical and preclinical studies indicates that females experience a faster progression to drug addiction and more severe addiction‐related health effects compared with males. Estradiol (E2) plays a critical role in these sex differences. Recently, we demonstrated that E2 significantly exacerbates adverse health effects, such as respiratory distress and weight loss, in ovariectomized (OVX) female rats during withdrawal from extended‐access fentanyl self‐administration. To uncover the mechanisms behind E2‐enhanced toxicity, we investigated proteomic changes in the plasma of fentanyl‐withdrawn OVX rats under both E2 and non‐E2 presentation conditions.Plasma samples were collected following extended‐access fentanyl self‐administration during protracted withdrawal, when adverse health effects were most pronounced. Using liquid chromatography coupled with electrospray ionization tandem mass spectrometry (LC‐ESI MS/MS) we conducted proteomic analysis in OVX rats comparing the effect of fentanyl withdrawal, with or without E2, to drug‐naïve control rats.We found a significant effect of fentanyl withdrawal on plasma proteomes within OVX rats. Fentanyl withdrawal was associated with a significant change in 15 plasma proteins including B‐factor, properdin (Cfb), apolipoprotein E (ApoE), complement 4, precursor (C4), C‐reactive protein (Crp), zinc‐alpha‐2‐glycoprotein precursor (Azgp1), and serine peptidase inhibitor 3L (Serinpa3l). The addition of E2 was associated with enhanced proteomic changes. Bioinformatic gene ontology enrichment analysis indicates that fentanyl withdrawal can disrupt the expression of proteins associated with immunity, lipid transport, and components of the extracellular matrix. We identify protein changes in plasma that may contribute to adverse health outcomes in females, with and without E2, during fentanyl withdrawal. These findings support the development of targeted strategies addressing health risks during opioid use disorder in women.

## 1 BACKGROUND

Despite higher rates of substance use disorder (SUD) and drug‐induced overdose deaths in men,^1^ studies show that women have an accelerated course from initial drug use to meeting criteria for SUD and/or seeking treatment for the disorder, a phenomenon also known as the telescoping effect.^2^ In addition, women experience more severe addiction‐related health effects including the development of drug‐associated diseases such as cirrhosis and hepatitis C.^3^ Similar sex differences have been observed in laboratory animals with results showing that female rats develop an addiction‐like phenotype after less drug exposure, and exhibit withdrawal symptoms more rapidly than males.^4^ In rats, this phenotype has been defined as the development of an enhanced motivation to obtain the drug, and an enhanced vulnerability to relapse.^5^, ^6^

Female rats exhibit a higher risk of developing severe adverse health consequences during fentanyl withdrawal, including prolonged weight loss, respiratory distress, and death,^7^, ^8^ but the biological mechanisms for this risk are not clear. Fentanyl is a potent synthetic opioid with high toxicity and risk to induce respiratory depression and overdose at relatively low doses.^9^ Women are vulnerable to the toxic effects of fentanyl, with differences in fentanyl metabolism between males and females.^10^, ^11^ In addition, ovarian hormones such as estradiol (E2) have been found to mediate the addictive as well as the toxic effects of opioid drugs such as fentanyl.^11^, ^12^, ^13^ Studies in OVX rats show that E2 loss diminishes drug self‐administration, including fentanyl,^7^, ^8^ and that E2 replacement facilitates the acquisition, escalation, and reinstatement of drug‐seeking behavior.^3^, ^14^, ^15^ Changes in E2 are also known to modulate the expression of several opioid receptors in the brain contributing to peptide and dopamine release within the reward pathway.^16^, ^17^

Using an ovariectomized (OVX) rat model of opioid addiction with fentanyl, we recently showed that E2 supplementation accelerates the development of addiction‐like behaviors during extended‐access self‐administration, as measured by an increase in drug intake and drug‐seeking and motivation behavior.^8^ E2 is also found to prolong the time‐course of physical dependence as measured by withdrawal‐induced weight loss and risk of developing adverse health effects that are observed during withdrawal.^8^ To identify factors that may contribute to fentanyl withdrawal and its associated adverse health effects in females, we performed proteomic analyses of plasma obtained from OVX female rats with and without E2 replacement and compared them with the drug‐naïve OVX control. Plasma was collected during protracted withdrawal following extended‐access fentanyl self‐administration, when the risk of adverse health effects is heightened.^8^ Samples were analyzed using liquid chromatography‐coupled electrospray ionization (LC‐ESI) tandem mass spectrometry (MS/MS). Our findings identify proteins that can contribute to the adverse health effects of drug use and withdrawal in females.

## 2 METHODS

### 2.1 Subjects

Adult OVX female Sprague–Dawley rats (*n* = 12) were purchased from Charles River Laboratory and arrived at our facility within 1 week of surgery. These animals were representative subsets of the Fentanyl+E2 (4 of 27) and Fentanyl alone (4 of 22) groups we used in our previous study on the impact of E2 on the development of addiction‐like features and adverse health effects.^8^ The rats received either a subcutaneous injection of E2 (5 μg/0.1 mL/day; Fentanyl+E2) or an equal volume of vehicle (corn oil; Fentanyl) at the same time (11:00 a.m.) 5 days a week.^8^ An additional cohort of vehicle‐treated OVX female rats (*n* = 4) given access to saline instead of fentanyl was used for comparison as drug‐naïve controls (Saline). All procedures were approved by the University of Virginia Animal Care and Use Committee and were conducted within the guidelines set by the National Institutes of Health.

### 2.2 Plasma collection

The procedures used for housing, surgical implantation of jugular catheters, fentanyl self‐administration training, extended‐access fentanyl self‐administration, and cue‐induced relapse testing are thoroughly described in our previous study.^8^ A 14‐day withdrawal period began following the last session wherein withdrawal‐induced changes in body weight relative to weight at the last session were assessed as a measure of general health and physical dependence. Trunk blood was collected the morning following the cue relapse test (between 10 a.m. and 12 p.m.) and separated into plasma and serum fractions as described.^18^, ^19^ Plasma was also collected from an additional cohort of OVX (Saline) rats that underwent similar procedures with training and extended access to saline instead of fentanyl.

### 2.3 Protein identification and quantification

Protein concentration for each plasma sample was determined using a Bradford assay, and treatment group samples for mass spectrometry analysis were created by aggregating an equal mass of protein from each plasma sample per condition similar to previous studies.^20^ A total of 50 μg of protein for each experimental condition was prepared for mass spectrometry following a protocol for denaturing, reducing, and alkylating proteins in 8 M urea, 48 mM dithiothreitol (DTT), and 9 mM iodoacetamide, respectively, in ammonium bicarbonate.^21^ Proteins were digested with trypsin at 37°C for 5 h, desalted with C18 ZipTip (Millipore), and then dehydrated in a SpeedVac and reconstituted in 0.1% formic acid before loading into the Exploris Orbitrap 480 equipped with an EASY‐nLC 1200 HPLC system (ThermoFisher Scientific, Waltham, MA, USA). Fragments were separated using a reverse‐phase PepMap RSLC 75 μm i.d. by 15 cm long with a 2 μm particle size C18 column (Thermo Fisher Scientific, Waltham, MA, USA), eluted with 0.1% formic acid and 80% acetonitrile at a flow rate of 300 nL/min. Peptides were fragmented by high‐energy collisions (HCD). Enabled filters included EASY‐IC internal mass calibration, monoisotopic precursor selection, and dynamic exclusion (20 s). All mass spectrometry samples were run in triplicate.

Proteins were identified by comparing the resulting spectra to predicted spectra in the *Rattus norvegicus* NCBI 2017 database using Proteome Discoverer *v*2.5 (ThermoFisher Scientific, Waltham, MA, USA) in data‐dependent acquisition (DDA). The mass tolerance for precursor ions was 5 parts per million (ppm) and the mass tolerance for fragment ions was set to 0.05 Da. The cutoff value for false discovery rate (FDR) was set to 1% for reporting peptide spectral matches (PSM). Proteins with 3 or more PSMs found in at least 2 of 3 replicates were analyzed. The average number of PSMs was used to create an abundance ratio between experimental groups: Fentanyl versus Saline; Fentanyl+E2 versus Saline; Fentanyl+E2 versus Fentanyl. A log_2_ transformed abundance ratio (fold‐change) was normalized using the equal median normalization method and assessed for significance with the bioconductor‐based DEqMS and Limma packages in the R Studio environment.^22^, ^23^ Proteins with a fold‐change of at least ±0.4 (log_2_ abundance ratio) and a *p*‐value <.05 were considered significant.

### 2.4 Data analysis

Repeated measures ANOVA was used to evaluate physical dependence, defined by changes in body weight over the 14‐day withdrawal period relative to pre‐withdrawal weights recorded on Day 1 following the final extended‐access session in the Fentanyl and Fentanyl+E2 groups. Saline controls were included to establish baseline (or “normal”) weight changes over this period. Within‐group one‐tailed *t*‐tests were conducted to determine if weight change significantly differed from the pre‐withdrawal baseline. Post‐hoc comparisons were Bonferroni‐corrected.

For proteomics analysis, the official gene symbols for the comprehensive set of significantly altered proteins from each comparison were uploaded to The Search Tool for the Retrieval of Interacting Genes/Proteins (STRING), and analyzed for functional enrichment.^24^ Analyses against Gene Ontology (GO) and Kyoto Encyclopedia of Genes and Genomics (KEGG) identified terms that are significantly enriched with the altered proteins found in each comparison. Official gene symbols for significantly altered proteins from each comparison were used as input for STRING.

## 3 RESULTS

### 3.1 An OVX rat model of fentanyl withdrawal for examining the physiological effects of E2

**FIGURE 1:**
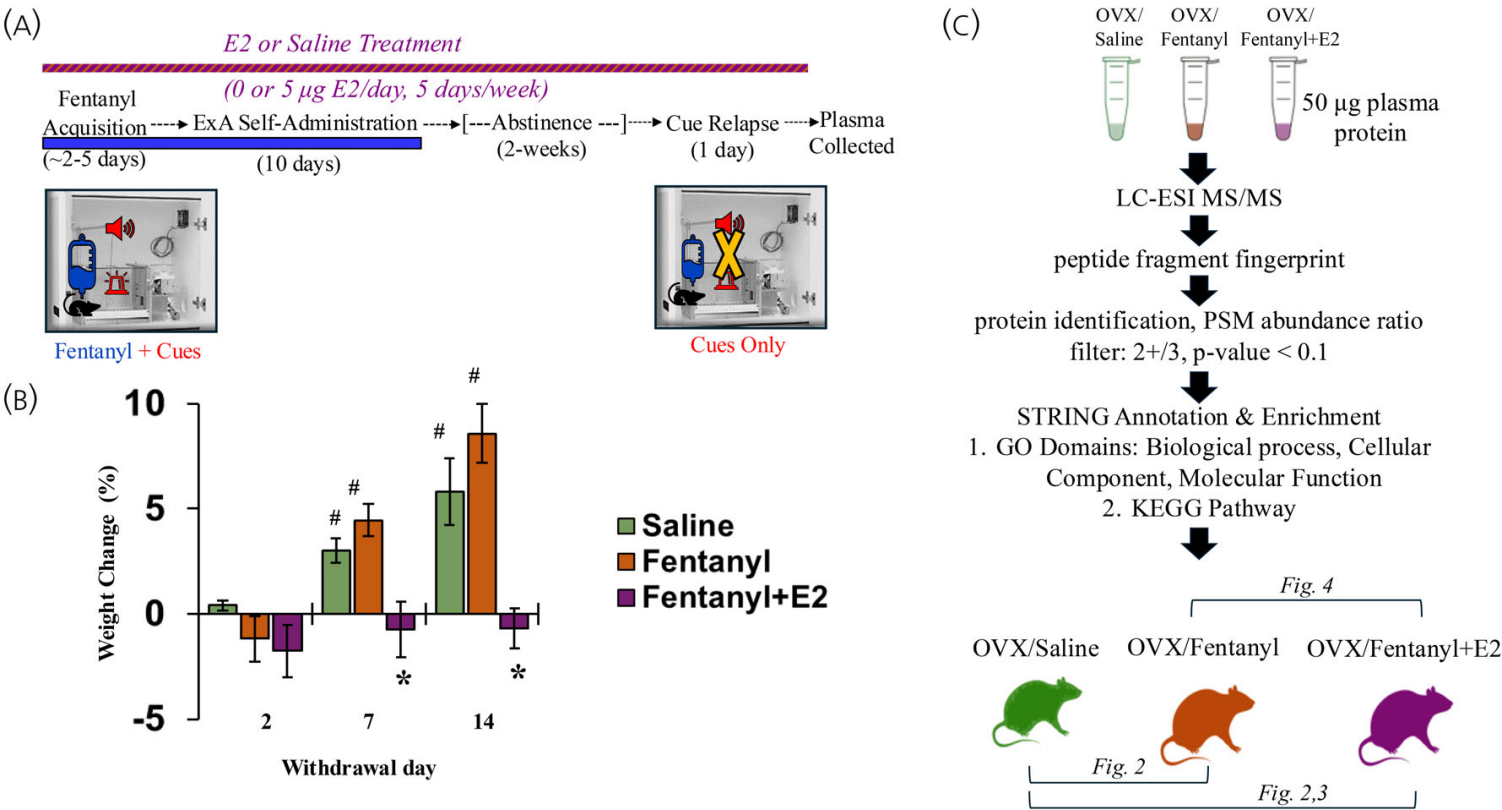
Summary of the study for testing the impact of E2 on fentanyl withdrawal within OVX female rats. (A) OVX female rats with and without E2 replacement were trained to self‐administer fentanyl under a fixed‐ratio 1 schedule (FR train; 0.25 μg/kg/infusion). Following acquisition, rats were given extended, intermittent‐access to fentanyl (0.25 μg/kg/infusion; 24 h/day, 2, 5‐min trials/h) for 10 days. (B) Physical dependence defined by withdrawal‐induced weight loss, was evaluated over a 14‐day withdrawal period. Weight remained significantly lower in rats self‐administering fentanyl while receiving E2 supplementation (Fentanyl+E2) than those not receiving E2 (Fentanyl) (*p < .05). Significant difference from both the Fentanyl and Saline groups; (^#^ *p* < .05). (C) 50 μg of plasma protein, collected after cue relapse, was analyzed using LC‐ESI MS/MS. Significantly altered proteins were identified using the abundance ratio of PSM and then bioinformatically analyzed in STRING. Multiple group comparisons were conducted across the datasets.

In this study, we used a recent OVX rat model of addiction with fentanyl that showed that E2 supplementation accelerates the development of addiction‐like behaviors, including escalated intake, enhanced drug‐seeking, and motivation for the drug.^8^ This model is summarized in Figure 1A. We find that OVX rats on fentanyl with E2 supplementation (Fentanyl+E2) or fentanyl alone (Fentanyl) exhibited a time‐course of physical dependence as measured by withdrawal‐induced weight loss. Specifically, a significant impact of E2 supplementation on the time‐course of physical dependence (group effect, *F*_2,9_ = 15.3, *p* < .001) was seen with the Fentanyl+E2 group exhibiting a prolonged course and failing to gain weight over the 14‐day withdrawal period compared with both the Fentanyl and Saline groups (*p*'s < .01) (Figure 1B). Weight gain in the Fentanyl+E2 group remained below that of both the Fentanyl and Saline groups (*p*'s < .05). We did not detect a change in fentanyl intake over the 10‐day extended‐access period across the Fentanyl and Fentanyl+E2 groups (Figure S1A). In addition, no significant group differences were observed for extinction or reinstatement responses, although 100% of the rats in the Fentanyl+E2 group (4 out of 4) reinstated responding compared with 75% in the Fentanyl group (3 out of 4) (Figure S1B).

### 3.2 Proteomic analysis of plasma from an OVX rat model of fentanyl withdrawal with and without E2 supplementation

We conducted proteomic analysis from 3 experimental fentanyl withdrawal groups with 4 rats per group: (1) OVX Fentanyl+E2; (2) OVX Fentanyl; (3) drug‐naïve or no‐fentanyl saline controls (Saline) (Figure 1). The Fentanyl+E2 group did not display a significant change in fentanyl intake over the 10‐day extended‐access period compared with Fentanyl. However, Fentanyl+E2 was associated with prolonged weight loss during withdrawal days (2 [W2], 7 [W7] and 14 [W14]) relative to their weight at the start of the time of withdrawal. Statistical analysis confirms the effect of Fentanyl+E2 on weight loss relative to Fentanyl (interaction of group by day, *F*_2,12_ = 8.7, *p* < .01; effect of group, *F*_1,6_ = 13.8, *p* < .01) (Figure 1B). We did not detect a significant group difference in extinction or reinstatement responses, although 100% of the rats in the Fentanyl+E2 group reinstated responding compared with 75% of the Fentanyl group (Figure 1B).

**FIGURE 2:**
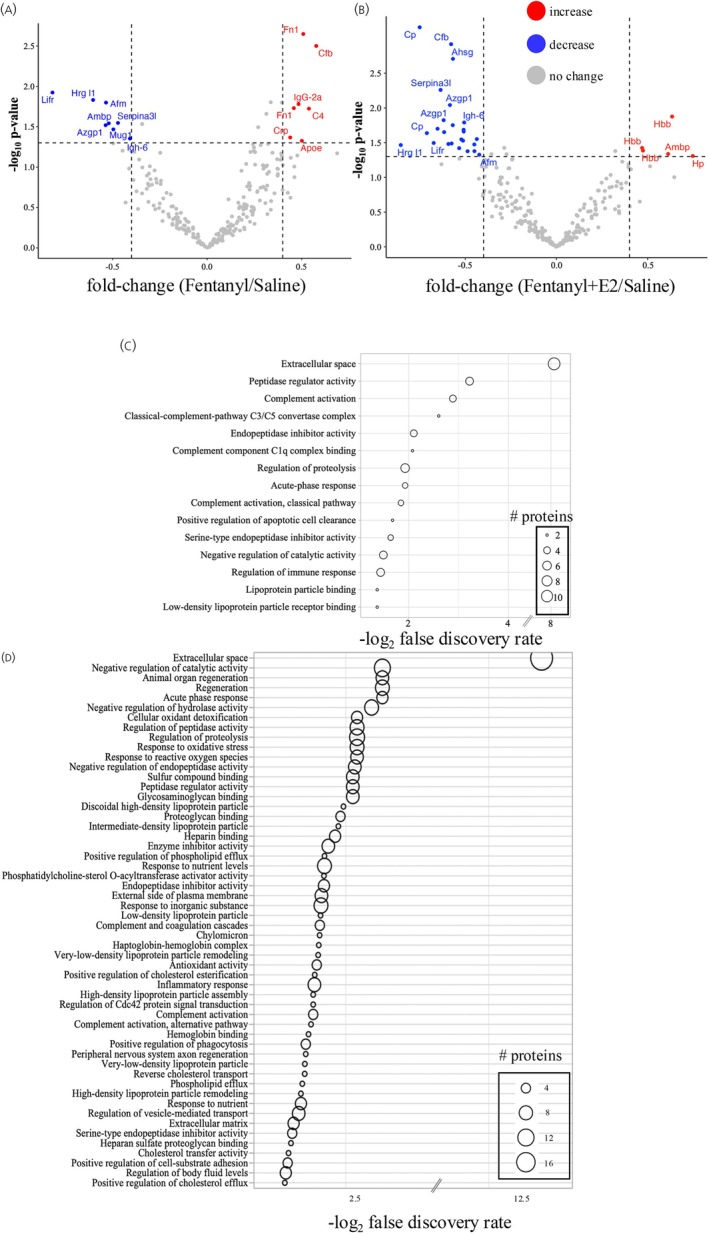
Proteomic results for Fentanyl and Fentanyl+E2 plasma fractions. (A) Volcano plot showing the distribution of significantly altered proteins within Fentanyl versus Saline. (B) Fentanyl+E2 versus Saline. (C) STRING enrichment analysis of terms associated with significantly altered proteins within the Fentanyl versus Saline. (D) Fentanyl+E2 versus Saline.

We used a quantitative MS proteomic strategy based on an analysis of PSM abundance ratio as previously described to examine changes in plasma proteins.^21^, ^25^ These abundance ratios reflect changes in the quantity of the identified proteins as measured by their PSM between the conditions. Direct PSM abundance ratio comparisons were performed between plasma proteins obtained from Fentanyl versus Saline or Fentanyl+E2 versus Saline where the Saline group was used as the drug‐naïve control. In addition, we examined proteomics changes between Fentanyl+E2 versus Fentanyl conditions from OVX rats (Figure 1C). Quantitative analysis was conducted using label‐free LC/ESI‐MS/MSMS as shown.^26^ A similar number of proteins were detected across all experimental conditions in the proteomic analysis. Specifically, we detected 442 total proteins within the Saline, 441 within the Fentanyl, and 536 within the Fentanyl+E2 plasma sample. Quantitative analyses of statistically significant proteins based on PSM measures revealed an effect of the drug condition on 15 proteins within the Fentanyl to Saline comparison (*p* < .05) (Figure 2A and Table 1). These significantly altered proteins included proteins associated with sepsis, such as B‐factor properdin (Cfb) and C‐reactive protein (Crp) (*p* < .05).^27^ Proteomic analysis also identified various isoforms amongst some proteins including hemoglobin beta subunit proteins (Hbb) and apolipoprotein E (ApoE). These isoforms are identified by their unique NCBI accession numbers along with their associated peptides in the MS (Table S1). An analysis of altered proteins between Fentanyl+E2 and Saline conditions identified 30 altered proteins (*p* < .05). PSM analysis also showed that Fentanyl+E2 was overwhelmingly associated with a decrease in the number of plasma proteins (Figure 2B and Table 2). The Fentanyl+E2 condition was associated with a reduction in known plasma carrier proteins including apolipoprotein a1 (ApoA) and ceruloplasmin (Cp) (*p* < .05) known for their involvement in lipid homeostasis.^28^, ^29^ The Fentanyl+E2 condition, however, was associated with an increase in Hbb and Ambp (alpha 1 microglobulin/bikunin), Hp (haptoglobin) (*p* < .05). This finding is consistent with the effect of E2 on increased blood oxygen count.^30^, ^31^

**TABLE 1:** Significantly altered proteins in OVX rats from Fentanyl versus Saline conditions.

Gene symbol	NCBI accession	Description	Fold change	*p*‐value	#Unique peptides
Cfb	CAE83972.1	B‐factor, properdin	0.5774	3.20 × 10^−3^	52
C4	P08649.3	Complement C4 (precursor)	0.5385	1.89 × 10^−2^	20
Fn1	XP_006245214.1	PREDICTED: fibronectin isoform ×3	0.5088	2.20 × 10^−3^	56
Apoe	EDM08152.1	Apolipoprotein E, isoform CRA_c	0.5014	4.72 × 10^−2^	50
IgG‐2a	AAH91257.1	LOC367586 protein	0.4839	1.66 × 10^−2^	0
Fn1	XP_006245213.1	PREDICTED: fibronectin isoform ×2	0.4588	1.86 × 10^−2^	56
Crp	AFA37859.1	C‐reactive protein	0.4396	4.29 × 10^−2^	4
Igh‐6	AAI03658.1	Igh‐6 protein, partial	−0.4086	4.42 × 10^−2^	1
Serpina3l	CAA34406.1	Serine (or cysteine) peptidase inhibitor, clade A, member 3 L	−0.4719	2.84 × 10^−2^	6
Mug1	AAA40630.1	Alpha‐1‐inhibitor III precursor, partial	−0.4964	3.41 × 10^−2^	6
Ambp	EDM10533.1	Alpha 1 microglobulin/bikunin, isoform CRA_a	−0.5206	2.87 × 10^−2^	17
Afm	EDL88554.1	Afamin, isoform CRA_b	−0.5353	1.59 × 10^−2^	39
Azgp1	Q63678.1	Zinc‐alpha‐2‐glycoprotein (precursor)	−0.5373	3.03 × 10^−2^	14
Hrg l1	AAG28417.1	Histidine‐rich glycoprotein	−0.6036	1.47 × 10^−2^	0
Lifr	NP_112310.1	Leukemia inhibitory factor receptor precursor	−0.8186	1.19 × 10^−2^	10

*Note*: Changes in individual proteins detected by MS analysis are shown as fold change (log_2_ of the abundance ratio measure between the conditions). A *p*‐value <.05 indicates statistical significance The number of unique peptides is the number of individual peptides associated with the protein.

**TABLE 2:** Significantly altered proteins in OVX rats from Fentanyl+E2 versus Saline conditions.

Gene symbol	NCBI accession	Description	Fold change	*p*‐value	#Unique peptides
Hp	AAA41348.1	Haptoglobin (Hp)	0.7473	4.92 × 10^−2^	24
Hbb	CAA33114.1	Beta‐globin	0.6339	1.33 × 10^−2^	4
Ambp	XP_008761981.1	PREDICTED: protein AMBP isoform ×1	0.6122	4.60 × 10^−2^	17
Hbb	NP_150237.1	Hemoglobin subunit beta‐1	0.4743	4.11 × 10^−2^	4
Hbb	AAA41309.1	Major beta‐hemoglobin	0.4695	3.77 × 10^−2^	2
Afm	EDL88554.1	Afamin, isoform CRA_b	−0.4236	4.72 × 10^−2^	39
Apoe	EDM08151.1	Apolipoprotein E, isoform CRA_b	−0.4368	2.81 × 10^−2^	11
Fn1	AAA41167.1	Fibronectin 2, partial	−0.45	4.19 × 10^−2^	19
Vtn	AAB01090.1	Vitronectin	−0.4509	3.36 × 10^−2^	15
A1bgl1	EDM16193.1	Similar to alpha 1B‐glycoprotein (predicted)	−0.4877	4.21 × 10^−2^	9
Igh‐6	AAI03658.1	Igh‐6 protein, partial	−0.5063	1.62 × 10^−2^	1
Ahsg	EDL78057.1	Alpha‐2‐HS‐glycoprotein, isoform CRA_b	−0.5089	2.19 × 10^−2^	6
Apoa1	NP_036870.1	Apolipoprotein A‐I preproprotein	−0.5094	2.06 × 10^−2^	47
Cfh	NP_569093.2	Complement factor H precursor	−0.5128	2.96 × 10^−2^	3
Ambp	EDM10533.1	Alpha 1 microglobulin/bikunin, isoform CRA_a	−0.5216	2.84 × 10^−2^	17
Vtn	AAP85374.1	Vitronectin (Aa1018)	−0.5337	3.80 × 10^−2^	15
Ahsg	AAP92571.1	Alpha‐2‐HS‐glycoprotein (Aa2‐066)	−0.5677	2.00 × 10^−3^	16
Mug1	AAA40630.1	Alpha‐1‐inhibitor III precursor, partial	−0.5688	1.77 × 10^−2^	17
Serpina3n	CAA31548.1	Contrapsin C‐term. (217 AA), partial	−0.5751	3.25 × 10^−2^	9
Cfb	NP_997631.2	Complement factor B precursor	−0.5779	1.20 × 10^−3^	52
Azgp1	XP_006249065.1	PREDICTED: zinc‐alpha‐2‐glycoprotein isoform ×1	−0.5845	9.10 × 10^−3^	14
Hbbl1	CAA34440.1	Protein product of hemoglobin subunit beta like 1	−0.5927	3.31 × 10^−2^	7
Fn1	AAA41168.1	Fibronectin 3, partial	−0.6162	2.23 × 10^−2^	18
Azgp1	Q63678.1	Zinc‐alpha‐2‐glycoprotein (precursor)	−0.6189	1.50 × 10^−2^	14
Serpina3l	CAA34406.1	Serine (or cysteine) peptidase inhibitor, clade A, member 3 L	−0.6356	5.50 × 10^−3^	6
Ambp	NP_037033.1	Protein AMBP precursor	−0.6524	1.99 × 10^−2^	20
Lifr	NP_112310.1	Leukemia inhibitory factor receptor precursor	−0.6735	3.20 × 10^−2^	10
Cp	AAA40915.1	Ceruloplasmin, partial	−0.7111	2.30 × 10^−2^	3
Cp	EDM01073.1	Ceruloplasmin, isoform CRA_c	−0.7504	7.00 × 10^−4^	36
Hrg l1	NP_001316829.1	Similar to histidine‐rich glycoprotein precursor	−0.854	3.42 × 10^−2^	8

*Note*: Changes in individual proteins detected by MS analysis are shown as fold change (log_2_ of the abundance ratio measure between the conditions). A *p*‐value <.05 indicates statistical significance The number of unique peptides is the number of individual peptides associated with the protein.

To gain insight into the biological implications of these proteomic findings, we explored the function of significantly altered proteins within both the Fentanyl and Fentanyl+E2 conditions. We used the STRING database of known and predicted information on physical and functional associations between proteins and signaling networks.^24^ We performed an enrichment analysis against Gene ontology (GO) and KEGG databases within STRING.^24^, ^32^ Enrichment analysis was first conducted on significantly altered proteins within the Fentanyl condition relative to the Saline. Our results show an effect of fentanyl on immune responses, including the complement cascade and lipoprotein regulation (Figure 2C). An enrichment analysis was also conducted on the Fentanyl+E2 proteomic dataset. As shown in Figure 2D, these data reveal an enrichment of proteins involved in the regulation of lipid synthesis and transport as well as immune responses. Our findings suggest that E2 increases the expression of immunity markers during fentanyl withdrawal. Our analysis of plasma proteins from the Fentanyl+E2 condition also reveals a modification in the expression of proteins that contribute to interactions between cells and their extracellular environment including heparan sulfate proteoglycan and glycosaminoglycan‐binding proteins such as vitronectin (Vtn) and fibronectin (Fn1).

**FIGURE 3:**
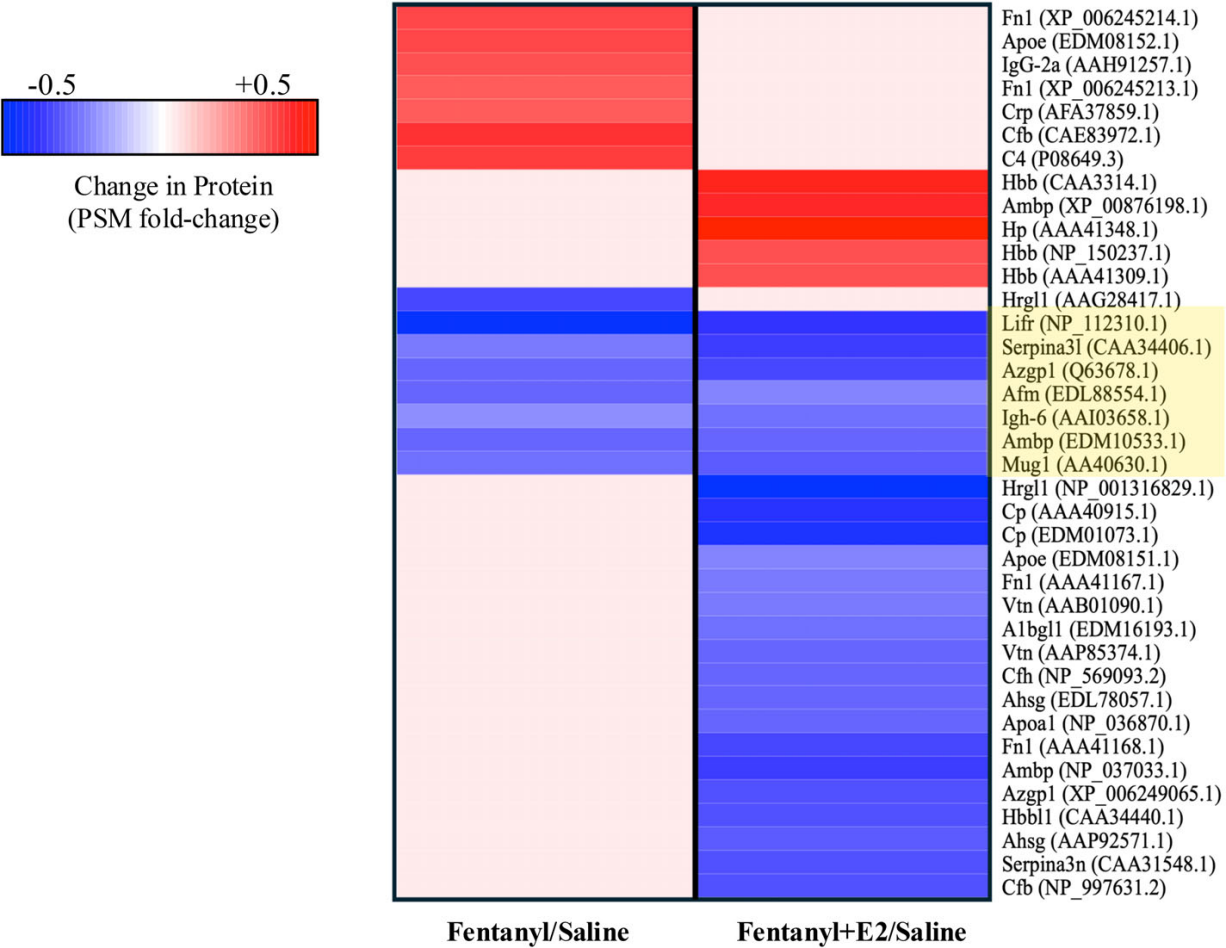
A heatmap showing fold‐changes in protein expression for proteins that are significantly altered within Fentanyl versus Saline and Fentanyl+E2 versus Saline comparisons. Common proteins between the two comparisons are boxed in yellow.

A comparison of significantly altered proteins within Fentanyl and Fentanyl+E2 conditions, each relative to Saline, is presented in Figure 3. This heatmap shows changes in the expression of proteins across the two proteomic datasets and highlights the area of overlap between them. Specifically, we find that 7 proteins are significantly altered in Fentanyl as well as Fentanyl+E2 proteomes (*p* < .05). These proteins are leukemia inhibitory factor receptor (Lifr), zinc‐alpha2‐glycoprotein (Azgp1), serine (or cysteine) peptidase inhibitor, clade A, member 3 L (Serpina3l), afamin isoform CRA_b (Afm), immunoglobulin heavy chain 6 (Igh‐6), alpha1 microglobulin/bikunin isoform CRA_a (Ambp) and alpha‐1‐inhibitor III precursor (Mug1). An analysis shows that these 7 proteins are decreased in their expression within the Fentanyl and Fentanyl+E2 plasma fractions relative to the Saline condition (*p* < .05).

### 3.3 A comparison of plasma proteomes between Fentanyl+E2 and fentanyl conditions in OVX rats

**FIGURE 4:**
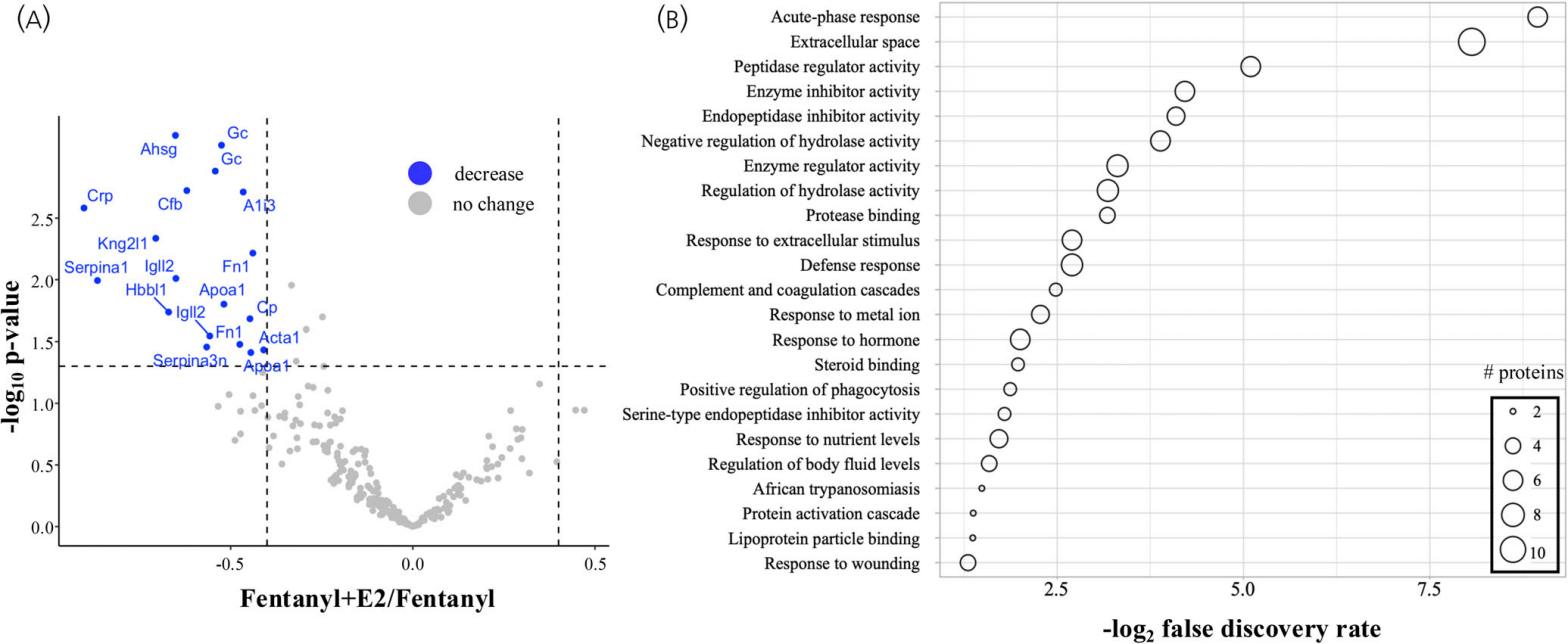
A comparison of Fentanyl+E2 and Fentanyl plasma proteomes. (A) Volcano plot showing the distribution of significantly altered proteins within Fentanyl+E2 and Fentanyl proteomes. (B) STRING enrichment analysis of terms associated with significantly altered proteins within Fentanyl+E2 and Fentanyl proteomes.

To further explore the specific impact of E2 supplementation on fentanyl withdrawal within the plasma proteome, we calculated PSM abundance ratios comparing Fentanyl+E2 and Fentanyl proteome datasets. Significantly altered proteins between Fentanyl+E2 and Fentanyl groups are presented in Table 3 and Figure 4. The volcano plot clearly shows that E2 supplementation is associated with a decrease in plasma proteins within the 18 significantly altered proteins identified within the dataset (Figure 4A). Bioinformatic analysis of these 18 proteins using STRING indicates enrichment in enzymatic activity (kininogen 2‐like 1 [Kng2l1], serine protease inhibitors 1 and 3n [Serpina1 and Serpina3n], alpha‐2 HS glycoprotein [Ahsg]), and in hormone as well as immune system function including acute‐phase responses and complement and coagulation cascades (Figure 4B). These results provide information on potential mechanisms underlying E2‐mediated effect during fentanyl withdrawal, and suggest a role for glycoprotein and lipid processes in adverse health effects in females.

**TABLE 3:** Significantly altered proteins in OVX rats from Fentanyl+E2 versus Fentanyl conditions.

Gene symbol	NCBI accession	Description	Fold change	*p*‐value	#Unique peptides
Acta1	EDL96714.1	Actin, alpha 1, skeletal muscle, isoform CRA_a	−0.4094	3.69 × 10^−2^	7
Fn1	XP_006245214.1	PREDICTED: fibronectin isoform ×3	−0.4391	6.10 × 10^−3^	56
Apoa1	NP_036870.1	Apolipoprotein A‐I preproprotein	−0.4446	3.87 × 10^−2^	47
Cp	EDM01073.1	Ceruloplasmin, isoform CRA_c	−0.447	2.07 × 10^−2^	36
A1i3	P14046.1	Alpha‐1‐inhibitor 3 precursor	−0.4655	1.90 × 10^−3^	32
Fn1	AAA41167.1	Fibronectin 2, partial	−0.4749	3.32 × 10^−2^	19
Apoa1	CAA25224.1	Preproapolipoprotein A‐I	−0.5185	1.58 × 10^−2^	42
Gc	AAA41082.1	Vitamin D‐binding protein precursor	−0.5252	8.00 × 10^−4^	59
Gc	NP_036696.2	Vitamin D‐binding protein precursor	−0.5422	1.30 × 10^−3^	60
Igll2	ABD65265.1	Immunoglobulin lambda light chain, partial	−0.5572	2.84 × 10^−2^	2
Serpina3n	CAA31548.1	Contrapsin C‐term. (217 AA), partial	−0.5658	3.50 × 10^−2^	9
Cfb	CAE83972.1	B‐factor, properdin	−0.6204	1.90 × 10^−3^	52
Igll2	ABD65264.1	Immunoglobulin lambda light chain, partial	−0.6503	9.80 × 10^−3^	1
Ahsg	AAP92571.1	Aa2‐066	−0.6515	7.00 × 10^−4^	16
Hbbl1	CAA34440.1	Protein product of hemoglobin subunit beta like 1 gene	−0.6702	1.83 × 10^−2^	7
Kng2l1	AAA41484.1	LMW K‐kininogen I precursor (AA at 138), partial	−0.7056	4.60 × 10^−3^	2
Serpina1	BAA00579.1	Protein product of serpin family A member 1 gene	−0.8655	1.02 × 10^−2^	10
Crp	AFA37866.1	CRP	−0.9026	2.60 × 10^−3^	5

*Note*: Changes in individual proteins detected by MS analysis are shown as fold change (log_2_ of the abundance ratio measure between the conditions). A *p*‐value <.05 indicates statistical significance The number of unique peptides is the number of individual peptides associated with the protein.

## 4 DISCUSSION

A goal of this study was to identify potential indicators of E2‐augmented toxicity during fentanyl withdrawal using OVX rats. E2 has known effects on the opioid system including the stimulation of endogenous opioid production (endorphins, enkephalins, and dynorphins), and the modulation of opioid receptor expression as well as signaling.^33^, ^34^, ^35^ For example, E2 treatment in OVX rodent models is shown to alter μ‐opioid receptor expression and internalization in the brain. ^17^, ^36^, ^37^ In addition, opioid receptors are expressed in immune cells^38^, ^39^ and interactions between E2 and opioid receptors can contribute to inflammatory conditions during cardiovascular disease.^35^, ^40^, ^41^ Our work highlights proteome responses to fentanyl withdrawal, and its potential for toxicity in females taking into account the role of E2 on adverse health effects including weight.^42^ Our findings may help explain why females exhibit more severe withdrawal effects.^8^

**FIGURE 5:**
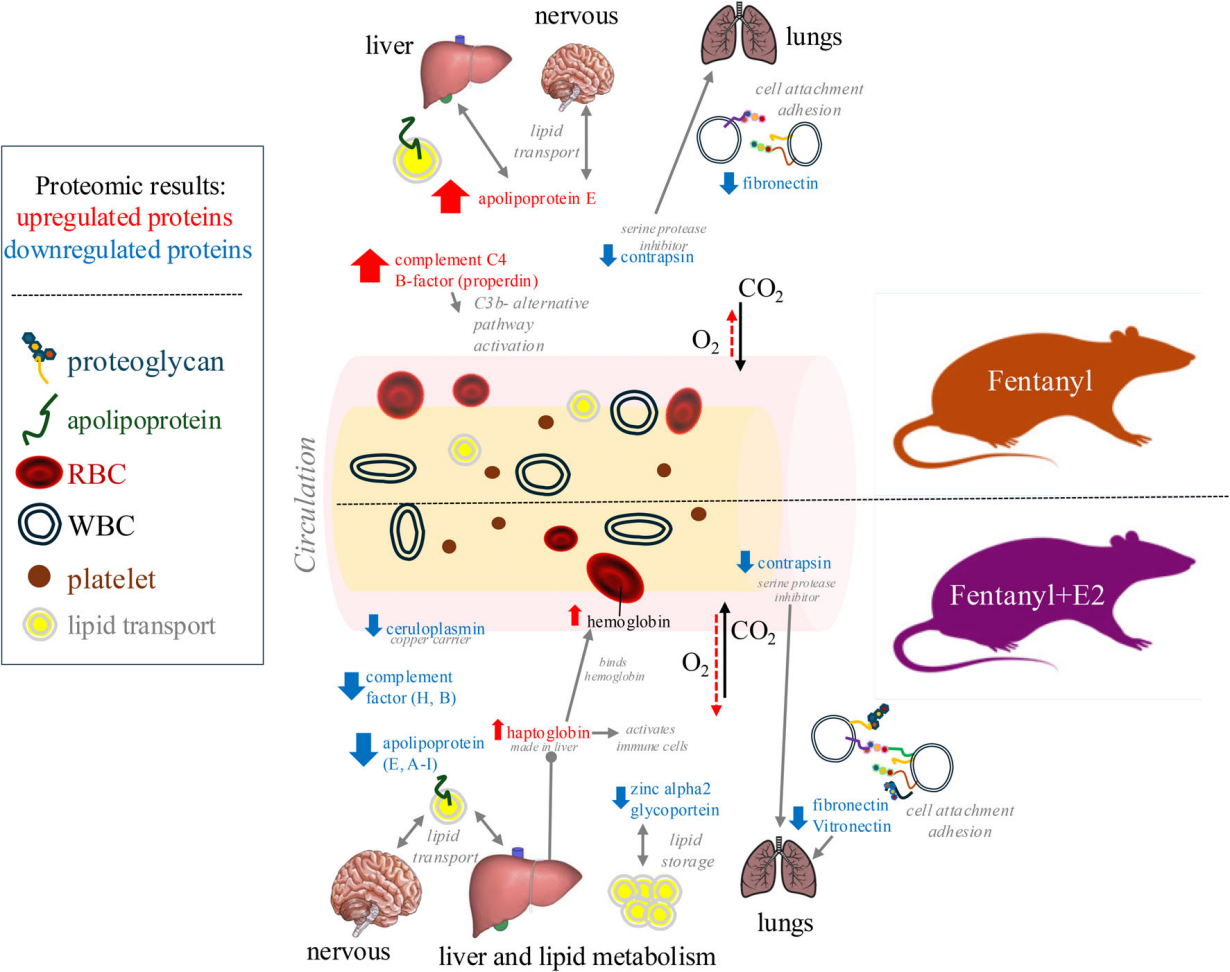
A schematic summary of proteomic findings showing an effect of fentanyl and E2 on primary pathways of immune system signaling and cellular regulation in the rat model.

Proteomic findings in this study  provide mechanistic information on interactions between E2 and fentanyl during withdrawal. A schematic in Figure 5 based on our proteomic findings highlights the potential for modifications amongst several interacting homeostatic systems involving glycoprotein activity, lipid transport, metabolism, and immune responses. We hypothesize that these proteomic changes reflect drug‐related modifications within multiple organs including the liver, brain, and immune cells such as lymphocytes, mast cells, and macrophages that are known to involve the function of the identified plasma proteins. Our results show that the composition of the plasma proteome can differ in OVX rats that are exposed to fentanyl with or without E2 supplementation. These findings are consistent with data showing that E2 supplementation potentiates markers of adverse health effects during fentanyl withdrawal, including weight loss and respiratory distress.^8^

### 4.1 Complement system and immune regulation

Exogenous opioids such as morphine and fentanyl have been found to impair the function of macrophages, natural killer cells, and T‐cells, and to weaken the gut barrier in vitro and in animal studies. The complement system (CS) includes more than 30 plasma proteins that provide defense against infection and remove toxic materials.^43^ Inherited CS deficiencies predispose individuals to bacterial infection and/or immune complex disease. Complement activation has been shown to be involved in morphine tolerance and withdrawal‐induced hyperalgesia in rodents.^44^ For example, the administration of complement protein C5a into rats was found to promote hyperalgesia and the expression of spinal antinociceptive tolerance to intrathecal morphine.^45^ Our study shows that complement proteins Cfb (B‐factor, properdin) and complement factor 4 (C4) are increased in OVX female rats during protracted withdrawal from fentanyl self‐administration (Fentanyl) suggestive of inflammation. In contrast, Cfb and Cfh are decreased in rats that receive E2 supplementation, suggesting that E2 may reverse the inflammatory effect during fentanyl withdrawal. The promotion of CS activity by a protein such as properdin may drive important changes in immune responses including cytokine production that can cause tissue damage.^46^, ^47^ Our findings suggest that E2 replacement modifies fentanyl withdrawal‐associated CS activation, a finding that is consistent with the impact of hormone replacement therapy (HRT) on women's immune function.^48^, ^49^ Interactions between E2 and fentanyl may modify CS immune responses in a manner that merits further study in both humans and animal models.

Additionally, our results demonstrate a significant increase in the expression of the immune system regulator C‐reactive protein (CRP) during withdrawal from fentanyl self‐administration. This fentanyl withdrawal effect on CRP was not observed in E2‐treated OVX rats, suggesting that E2 offsets the effect of fentanyl withdrawal on this protein. CRP is mostly synthesized in liver hepatocytes but is also made within muscle cells, macrophages, lymphocytes, and adipocytes.^50^, ^51^, ^52^ CRP is shown to bind to lysophosphatidylcholine on the surface of dead or dying cells and can activate the CS. Interestingly, HRT has also been shown to influence circulating levels of CRP within women, and CRP blood concentrations have been used as markers of infection, cardiovascular disease, and immune responses.^53^, ^54^ These processes may contribute to higher morbidity rates in earlier studies of fentanyl effect on rats in the presence of E2.^8^

E2 supplementation was found to shift the expression of fibronectin within the plasma from being increased within the Fentanyl group to being decreased within the Fentanyl+E2 group. Fibronectin is a glycoprotein that is a critical component of the extracellular matrix and participates in cell structure and motility.^55^ Plasma fibronectin levels have been shown to correlate with immune signaling responses in various addiction models including alcoholic liver disease.^55^, ^56^ Our proteomic analysis highlights an effect of fentanyl and E2 on fibronectin expression. Similarly, proteomic analysis underscores the interactive effect of Fentanyl+E2 on the protein Serpina1 (aka alpha‐1 antitrypsin), which is a serine protease inhibitor (serpin) that participates in the control of various enzymes including digestive enzymes as well as white blood cell inflammatory regulators such as neutrophil elastase.^57^

### 4.2 Glycoprotein and lipid regulation

Our results point to molecular changes that highlight interactions between fentanyl and E2 within the plasma glycoproteome. Notably, we find changes in proteins that are involved in sugar‐based signaling in various cell types including immune cells. For example, our proteomic results indicate an effect of Fentanyl+E2 on glycosaminoglycan and heparan sulfate proteoglycan‐binding proteins. Cell surface glycosylation is important for the proper function of innate and adaptive immune responses and plays a crucial role in CS activity,^58^, ^59^ which is found to be modified within our proteomic data. Various drugs of abuse, including alcohol and opioids, have been shown to modify glycosylation in various cell types through changes in the expression of glycosylation‐regulating enzymes and binding proteins, as well as the elimination of glycoconjugates in the extracellular environment.^60^, ^61^ Such glycoproteins that were found to be modified by Fentanyl in our study include histidine‐rich glycoprotein (Hrg l1) and zinc alpha 2 glycoprotein (Azgp1), which are important in lipid metabolism and the regulation of body weight.^62^, ^63^ Changes in protein glycosylation are also implicated in cancer, cardiovascular disease, and respiratory as well as metabolic conditions.^64^, ^65^, ^66^

Apolipoproteins (Apo) have been shown to carry fentanyl in blood.^67^ Our proteomic analysis indicates that fentanyl modifies the expression of multiple Apo proteins that seem to be differentially impacted when fentanyl is administered alone or in combination with E2. Specifically, ApoA and ApoE (isoform CRA_b) are found to be significantly decreased in the Fentanyl+E2 group, while in Fentanyl alone we find that ApoE level (isoform CRA_c) is increased. These two rat ApoE isoforms appear to differ by 172 amino acids, but nothing is known about functional differences between them. ApoE is a conserved mammalian lipoprotein involved in lipid homeostasis and cholesterol transport through the regulation of phospholipid cholesteryl and ester triacylglycerol (TAG) transport across the cell membrane.^68^, ^69^ It is primarily synthesized in the liver and in circulating macrophages, but is also found in glia within the brain and spinal cord where it has been shown to participate in neuron survival and health.^70^ These changes in ApoE within our study suggest an important interaction between E2 and fentanyl in lipid transport and signaling within various systems including nervous tissue and immune cells.

## 5 CONCLUSION

Interactions between fentanyl and the immune system are documented in the literature and shown to impact inflammatory regulation during pain responses in humans and rodent models.^71^, ^72^ These studies, however, are largely conducted in male rodents with less known on the effects of fentanyl in females. Our proteomic analysis begins to address this discrepancy by identifying proteins and pathways that are impacted by fentanyl withdrawal in OVX females with and without E2 replacement based on our recently developed rat model.^8^ Thus while the current analysis is based on the detection of proteins within plasma future studies can extend this by assessing changes in effected organ systems inlcuding brain and spinal cord and provide. insight for the development of clinical interventions for opioid use disorder.

## AUTHOR CONTRIBUTIONS

**Patricia Sinclair:** Conceptualization; methodology; investigation; formal analysis; data curation; writing – review and editing. **Navdeep S. Dhanjal:** Conceptualization; methodology; investigation; formal analysis; data curation. **E. Blair Towers:** Investigation; methodology. **Dr. Wendy Lynch:** Conceptualization; formal analysis; writing – review and editing; project administration; resources. **Nadine Kabbani:** Conceptualization; formal analysis; writing – review and editing; project administration; resources.

## CONFLICT OF INTEREST STATEMENT

The authors declare no conflict of interest.

## PEER REVIEW

The peer review history for this article is available at https://www.webofscience.com/api/gateway/wos/peer‐review/10.1111/jne.70033.

## Supporting information


**Data S1.** Supporting information.

## Data Availability

Data sharing is not applicable to this article as no new data were created or analyzed in this study.
