# Supplementary material for: Proteomic analysis of plasma proteins during fentanyl withdrawal in ovariectomized female rats with and without estradiol
Source: J Neuroendocrinol. 2025 Apr 20;37(8):e70033. doi: 10.1111/jne.70033 (PMC12358201; doi:10.1111/jne.70033)
Supplement: Supplementary file 1 — Data S1. Supporting information. [file JNE-37-e70033-s001.docx]

**Supplementary Material**

**Supplementary Figure 1. A)** A comparison of fentanyl intake over the 10-day extended-access period between Fentanyl and Fentanyl+E2 conditions. **B)** Vulnerability to relapse was assessed on withdrawal day 15 using a within-session extinction/cue-induced relapse procedure. No group differences in extinction or reinstatement responses were observed between Fentanyl+E2 and Fentanyl conditions. Repeated measures ANOVA shows no significant difference between any of the conditions (p > 0.05).

**A**

**B**


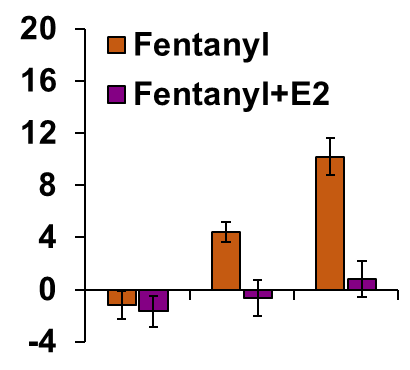

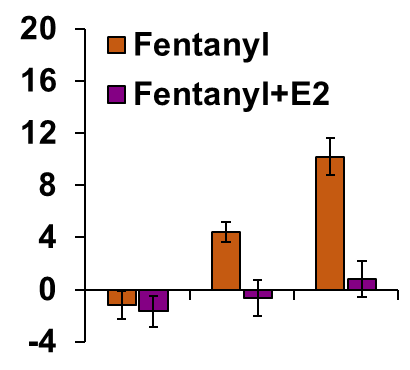

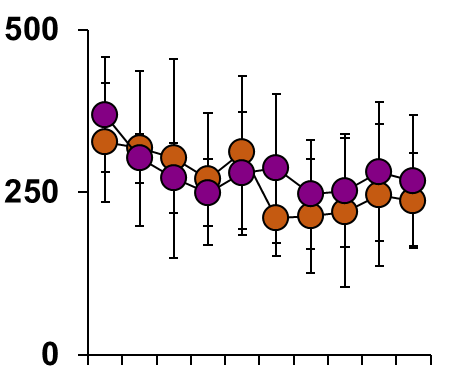


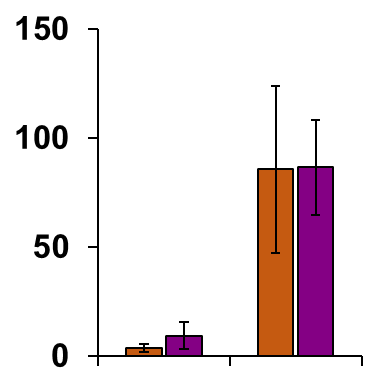


**Extinction Reinstate**


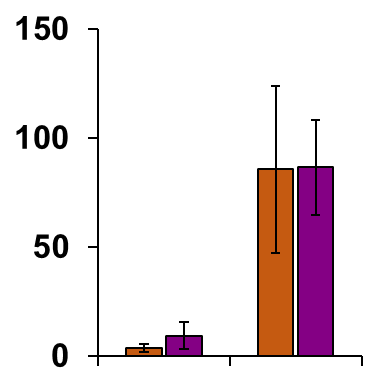


**1 2 3 4 5 6 7 8 9 10**

**Day of extended access**

**Infusions**

**Responses**

**Supplementary Table 1**. Unique peptides amongst protein isoforms identified by NCBI accession numbers.

| **Gene Symbol** | | **NCBI Accession** | | **Description** |
| --- | --- | --- | --- | --- |
| **Cfb** | | **NP_9976312/ CAE839721** | | **Complement factor b, precursor/B-factor properidin** |
| QQLVPSYAR  RCLANLIEKVASYGVKPR  LKYSQTLRPICLPCTEGTTR  LKDEDLGFL  STGSWSVLQTRDQKIVK  VLVRVSEER  VASYGVKPR  VASEVVTPR  VKVASEVVTPR  VGSQYRLEDTVTYHCSR  YGLVTYATVPK  YGLVTYATVPKVLVR  YGLVTYATVPKVLVRVSEER  YSQTLRPICLPCTEGTTR  SSDADWVTEKLNQISYEDHKLK  SSDADWVTEKLNQISYEDHK  STGSWSVLQTR[D]  SLGLCGMVWEHQK  TCQENGRWDGQTAICDDGAGYCPNPGIPIGTRK  TCKSTGSWSVLQTR[D]  TKLKYSQTLRPICLPCTEGTTR  DLEIEEVLFHPNYDINGK  DLEIEEVLFHPNYDINGKK  CPRPQDFENGEFWPR  DFHINLFQVLPWLK  DFHINLFQVLPWLKEK  ALFVSEEGK  ALFVSEEGKK  ALFVSEEGKKLTR  ALRLPQTATCK  AIRCPRPQDFENGEFWPR  AEGISEFYDYDVALIKLK  CLANLIEK  CLANLIEKVASYGVKPR  ASCERDATKAQGYEK  ALRLPQTATCK  AIRCPRPQDFENGEFWPR  KAEGISEFYDYDVALIK  KAEGISEFYDYDVALIKLK  GLVLRGSQQRR[C]  LEDTVTYHCSR  KVGSQYRLEDTVTYHCSR  GGDYYKQPWQAK  FLCTGGVDPYADPNTCKGDSGGPLIVHKR  GGSFQLLQDGQALEYLCPSGFYPYPVQTR  DMEDLENVFYK  STGSWSVLQTRDQK  WDGQTAICDDGAGYCPNPGIPIGTR  GLVLRGSQQR  TCKSTGSWSVLQTRDQK  STGSWSVLQTRDQKIVK  RQQLVPSYAR | | | | |
| **Cfh** | | **NP_5690932** | | **complement factor H precursor** |
| NGFYPATR  NGFYPATRSPVSK[C]  NGFYPATRSPVSKCTITGWIPAPR | | | | |
| **Apoe** | | **EDM08152.1** | | **apolipoprotein E, isoform CRA_c** |
| \| AGAQEGAERGVSAIR \| \| --- \| \| AGAQEGAERGVSAIRER \| \| AQALSDRIR \| \| AQALSDRIRGR \| \| ARLAKEVQAAQAR \| \| AYKKELEEQLGPVAEETR \| \| DRLEEVR \| \| DRLEEVREQMEEVR \| \| ELEEQLGPVAEETR \| \| ELEEQLGPVAEETRAR \| \| EQMEEVR \| \| EQMEEVRSKMEEQTQQIR \| \| ERLGPLVEQGR \| \| ERLGPLVEQGRQR \| \| GRLEEVGNQAR \| \| GRLEEVGNQARDR \| \| GWFEPLVEDMQR \| \| IKGWFEPLVEDMQR \| \| IKGWFEPLVEDMQRQWANLMEK \| \| IQASVATNSIASTTVPLENQ \| \| IRGRLEEVGNQAR \| \| LAKEVQAAQAR \| \| LAVYKAGAQEGAERGVSAIR \| \| LEEVGNQAR \| \| LEEVGNQARDRLEEVR \| \| LEEVREQMEEVR \| \| LGADMEDLR \| \| LGADMEDLRNR \| \| LGADMEDLRNRLGQYR \| \| LGPLVEQGR \| \| LGPLVEQGRQR \| \| LGQYRNEVNTMLGQSTEELR \| \| LGQYRNEVNTMLGQSTEELRSR \| \| LMRDADDLQKR \| \| LQAEIFQAR \| \| LSTHLRKMR \| \| MEEQTQQIRLQAEIFQAR \| \| NEVNTMLGQSTEELR \| \| NEVNTMLGQSTEELRSR \| \| QRTANLGAGAAQPLR \| \| QRTANLGAGAAQPLRDR \| \| QWANLMEK \| \| QWANLMEKIQASVATNSIASTTVPLENQ \| \| RLAVYKAGAQEGAER \| \| SKMEEQTQQIR \| \| SKMEEQTQQIRLQAEIFQAR \| \| TANLGAGAAQPLR \| \| TANLGAGAAQPLRDR \| \| TANLGAGAAQPLRDRAQALSDR \| | | | | |
| **Apoe** | | **EDM08151.1** | | **apolipoprotein E, isoform CRA_b** |
| \| GWFEPLVEDMQR \| \| --- \| \| IKGWFEPLVEDMQR \| \| IKGWFEPLVEDMQRQWANLMEK \| \| IQASVATNSIASTTVPLENQ \| \| LQAEIFQAR \| \| MEEQTQQIR \| \| MEEQTQQIRLQAEIFQAR \| \| QWANLMEK \| \| QWANLMEKIQASVATNSIASTTVPLENQ \| \| SKMEEQTQQIR \| \| SKMEEQTQQIRLQAEIFQAR \| | | | | |
| **Hbb** | | **CAA33114.1** | | **beta-globin** |
| AAVNGLWGK  AAVNGLWGKVNPDDVGGEALGR  AAVNGLWGKVNPDDVGGEALGRLLVVYPWTQR  AHGKKVINAFNDGLK  LHLHVDPENFR  LLGNMIVIVLGHHLGK  LLVVYPWTQR  LLVVYPWTQRYFDSFGDLSSASAIMGNPK  LLVVYPWTQRYFDSFGDLSSASAIMGNPKVK  VINAFNDGLKHLDNLK  VNPDDVGGEALGR  VNPDDVGGEALGRLLVVYPWTQR  VVAGVASALAHKYH   \| YFDSFGDLSSASAIMGNPK \| \| --- \| \| YFDSFGDLSSASAIMGNPKVK \| \| YFDSFGDLSSASAIMGNPKVKAHGK \| | | | | |
| **Hbb** | | **NP_150237.1** | | **Hemoglobin subunit beta-1** |
| VNPDDVGGEALGR  VNPDDVGGEALGRLLVVYPWTQR  AAVNGLWGKVNPDDVGGEALGR  AAVNGLWGKVNPDDGGEALGRLLVVYPWTQR | | | | |
| **Hbb** | | **AAA41309.1** | | **major beta-hemoglobin** |
| AHGKKVINAFNDGLK  EFTPCAQAAFQK  EFTPCAQAAFQKVVAGVASALAHKYH  LHVDPENFR  LLGNMIVIVLGHHLGK  LLGNMIVIVLGHHLGKEFTPCAQAAFQK  LLVVYPWTQRYFDSFGDLSSASAIMGNPK  LLVVYPWTQRYFDSFGDLSSASAIMGNPKVK  LVVYPWTQR  VINAFNDGLKHLDNLK  VNPDDVGGEALGR  VNPDDVGGEALGRLLVVYPWTQR  VVAGVASALAHKYH  YFDSFGDLSSASAIMGNPK  YFDSFGDLSSASAIMGNPKVK  YFDSFGDLSSASAIMGNPKVKAHGK | | | | |
| **Hbbl1** | | **CAA34440.1** | | **protein product of hemoglobin subunit beta like 1** |
| RLLVVYPWTQRYFSKFGDLSSASAIMGNPQVKA  RLLVVYPWTQRYFSKF  RYFSKFGDLSSASAIMGNPQVKA  KATVSGLWGKV  KATVSGLWGKVNPDNVGAEALGRL  KFGDLSSASAIMGNPQVKAHGKKV  KFGDLSSASAIMGNPQVKAHGKK | | | | |
| **Cp** | | **AAA40915.1** | | **ceruloplasmin, partial** |
| \| TYIWQIPER \| \| --- \| \| TKSSTVAPTLPGEVR \| \| SSTVAPTLPGEVR \| | | | | |
| **Cp** | | **EDM01073.1** | | **ceruloplasmin, isoform CRA_c** |
| FTKENEGTYYGPDGRSSK  EFTDSTFREQVKR  FTKENEGTYYGPDGR  ENEGTYYGPDGR  SGAGTEDSPCIPWAYYSTVDRVK  TYIWQIPERSGAGTEDSPCIPWAYYSTVDR  TYIWQIPERSGAGTEDSPCIPWAYYSTVDRVK  MYYSGVDLTKDIFTGLIGPMK  DLYSGLIGPLIVCR  VKDLYSGLIGPLIVCR  DLYSGLIGPLIVCRK  ETFTYEWTVPKEMGPTYADPVCLSK  EMGPTYADPVCLSK  TYIWQIPER  VKDLYSGLIGPLIVCRK  TKSSTVAPTLPGEVR  SSKQASHVAPKETFTYEWTVPK  SSTVAPTLPGEVR  AEEEHLGILGPLIHADVGDKVK  RAEEEHLGILGPLIHADVGDKVK  MYYSGVDLTKDIFTGLIGPMKICK  SGGTEDSPCIPWAYYSTVDR  NMASRPYSIHAHGVK  VVYREFTDSTFR  GQYREFTDSTFR  MYYSGVDLTK  DTANLFPHK  GSLLADGR  DIFTGLIGPMKICK  ICKKGSLLADGR  VVYREFTDSTFREQVK  EFTDSTFREQVK | | | | |
| **Gc** | | **AAA41082.1** | | **Vitamin D-binding protein precursor** |
| EVVSLTEECCAEGADPNCYDTR  VCSQYAAYGK  QLTSFIEKGQEMCADYSENTFTEYK  CCSINSPPRYCSSQIDAEMRDILQS  RTQVPEVFLSKVLDTTLK  VCSQYAAYGKEK  LQMKQLSLLTTMSNRVCSQYAAYGK  QLTSFIEKGQEMCADYSENTFTEYKK  MPNASPEELADMVAK  VLDTTLKTLRECCDTQDSVSCFSTQSPLMK  DLCGQSATQAMDQYTFELSRR  MPNASPEELADMVAKHSDFASK  VLDTTLK  TQVPEVFLSKVLDTTLK  LAQKVPTANLEDVLPLAEDLTEILSR  GFADQFLFEYSSNYGQAPLPLLVGYTK  MSHLIKLAQKVPTANLEDVLPLAEDLTEILSR  TQVPEVFLSKVLDTTLKTLR  CCKSTSEDCMAR  DDFRSLSLILYSR  DLCGQSATQAMDQYTFELSR  TKMPNASPEELADMVAK  TSELSIKSCESDAPFPVHPGTSECCTKEGLER  TLRECCDTQDSVSCFSTQSPLMKR  TSELSIKSCESDAPFPVHPGTSECCTK  CCSINSPPRYCSSQIDAEMR  VCQELSTLGKDDFR  SYLSMVGSCCTSAKPTVCFLKER  ECCDTQDSVSCFSTQSPLMKR  TQVPEVFLSK  QLSLLTTMSNR  DKVCQELSTLGKDDFR  QLSLLTTMSNRVCSQYAAYGKEK  KFPSSTFEQVSQLVK  VLDTTLKTLR  YCSSQIDAEMR  SCESDAPFPVHPGTSECCTK  ELPEHTLKICGNLSK  SYLSMVGSCCTSAKPTVCFLK  RTQVPEVFLSK  EVVSLTEECCAEGADPNCYDTRTSELSIK  SLSLILYSR  GQEMCADYSENTFTEYKKK  TKMPNASPEELADMVAKHSDFASK  SCESDAPFPVHPGTSECCTKEGLER  LQMKQLSLLTTMSNR  ECCDTQDSVSCFSTQSPLMK  KFPSSTFEQVSQLVKEVVSLTEECCAEGADPNCYDTR  TLRECCDTQDSVSCFSTQSPLMK  YCSSQIDAEMRDILQS  QLTSFIEK  LRTKMPNASPEELADMVAK  ELPEHTLKICGNLSKK  VPTANLEDVLPLAEDLTEILSR  TSELSIK  VCSQYAAYGKEKSR  HSDFASKCCSINSPPRYCSSQIDAEMR  ELPEHTLK | | | | |
| **Gc** | **NP_036696.2** | | **Vitamin D-binding protein precursor** | |
| EVVSLTEECCAEGADPNCYDTR  VCSQYAAYGK  QLTSFIEKGQEMCADYSENTFTEYK  CCSINSPPRYCSSQIDAEMRDILQS  RTQVPEVFLSKVLDTTLK  VCSQYAAYGKEK  KLCMAALSHQPQEFPAYVEPTNDEICEAFR  LQMKQLSLLTTMSNRVCSQYAAYGK  QLTSFIEKGQEMCADYSENTFTEYKK  MPNASPEELADMVAK  VLDTTLKTLRECCDTQDSVSCFSTQSPLMK  DLCGQSATQAMDQYTFELSRR  MPNASPEELADMVAKHSDFASK  VLDTTLK  TQVPEVFLSKVLDTTLK  LAQKVPTANLEDVLPLAEDLTEILSR  GFADQFLFEYSSNYGQAPLPLLVGYTK  MSHLIKLAQKVPTANLEDVLPLAEDLTEILSR  TQVPEVFLSKVLDTTLKTLR  CCKSTSEDCMAR  CCSINSPPR  DDFRSLSLILYSR  DLCGQSATQAMDQYTFELSR  TKMPNASPEELADMVAK  TSELSIKSCESDAPFPVHPGTSECCTKEGLER  TLRECCDTQDSVSCFSTQSPLMKR  TSELSIKSCESDAPFPVHPGTSECCTK  CCSINSPPRYCSSQIDAEMR  VCQELSTLGKDDFR  SYLSMVGSCCTSAKPTVCFLKER  ECCDTQDSVSCFSTQSPLMKR  TQVPEVFLSK  QLSLLTTMSNR  DKVCQELSTLGKDDFR  QLSLLTTMSNRVCSQYAAYGKEK  KFPSSTFEQVSQLVK  VLDTTLKTLR  YCSSQIDAEMR  SCESDAPFPVHPGTSECCTK  ELPEHTLKICGNLSK  SYLSMVGSCCTSAKPTVCFLK  RTQVPEVFLSK  EVVSLTEECCAEGADPNCYDTRTSELSIK  SLSLILYSR  GQEMCADYSENTFTEYKKK  TKMPNASPEELADMVAKHSDFASK  SCESDAPFPVHPGTSECCTKEGLER  LQMKQLSLLTTMSNR  ECCDTQDSVSCFSTQSPLMK  KFPSSTFEQVSQLVKEVVSLTEECCAEGADPNCYDTR  TLRECCDTQDSVSCFSTQSPLMK  YCSSQIDAEMRDILQS  QLTSFIEK  LRTKMPNASPEELADMVAK  ELPEHTLKICGNLSKK  VPTANLEDVLPLAEDLTEILSR  TSELSIK  VCSQYAAYGKEKSR  HSDFASKCCSINSPPRYCSSQIDAEMR | | | | |
